# Modelling of Web-Crippling Behavior of Pultruded GFRP I Sections at Elevated Temperatures

**DOI:** 10.3390/polym14235313

**Published:** 2022-12-05

**Authors:** Lingfeng Zhang, Qianyi Li, Ying Long, Dafu Cao, Kai Guo

**Affiliations:** 1 College of Civil Science and Engineering, Yangzhou University, Yangzhou 225127, China; 2 Department of Architecture, Built Environment and Construction Engineering, Politecnico di Milano, Piazza Leonardo da Vinci 32, 20133 Milano, Italy

**Keywords:** fiber-reinforced polymer, pultrusion, elevated temperature, web crippling, end-two-flange (ETF), end bearing with ground support (EG)

## Abstract

The concentrated transverse load may lead to the web crippling of pultruded GFRP sections due to the lower transverse mechanical properties. Several investigations have been conducted on the web-crippling behavior of the GFRP sections under room temperature. However, the web-crippling behavior is not yet understood when subjected to elevated temperatures. To address this issue, a finite element model considering the temperature-dependent material properties, Hashin failure criterion and the damage evolution law are successfully developed to simulate the web-crippling behavior of the GFRP I sections under elevated temperatures. The numerical model was validated by the web-crippling experiments at room temperature with the end-two-flange (ETF) and end bearing with ground support (EG) loading configurations. The developed model can accurately predict the ultimate loads and failure modes. Moreover, it was found that the initial damage was triggered by exceeding the shear strength at the web-flange junction near the corner of the bearing plate and independent of the elevated temperatures and loading configurations. The ultimate load and stiffness decreased obviously with the increasing temperature. At 220 °C, the ultimate load of specimens under ETF and EG loading configurations significantly decreased by 57% and 62%, respectively, whereas the elastic stiffness obviously reduced by 87% and 88%, respectively.

## 1. Introduction

Pultruded glass fiber-reinforced polymer (GFRP) sections have been extensively used as structural members in civil engineering applications due to their advantages such as lightweight, high strength, excellent durability, high design flexibility and good assembly [1,2,3]. The pultruded GFRP sections generally exhibited orthotropic mechanical behavior due to their inherent fiber architectures. The strengths and moduli in the longitudinal direction (parallel to glass rovings) were much higher than those in the transverse direction. As a result, the pultruded GFRP sections are prone to web crippling when subjected to transverse concentrated loads [4,5,6]. The web crippling was well understood in the case of metallic structures, which are significantly affected by the depth-thickness ratio (slenderness) of the web, section type and material strength. The web crippling of metallic structures usually comprises the web buckling and web yielding [7]. In contrast, the web-crippling of the pultruded GFRP section commonly consists of web buckling and web crushing [4,5,8,9]. Significant processes have been achieved in the investigation of the web crippling behavior of metallic structures. Four web-crippling loading configurations for cold-formed stainless steel hollow sections, end-one-flange (EOF), end-two-flange (ETF), interior-one-flange (IOF) and interior-two-flange (ITF), were specified in the existing design standards/specifications including the ASCE specification [10], AS/NZS 4673 [11] and Eurocode3 [12]. These loading configurations were adopted by several researchers to investigate the web-crippling behavior of pultruded GFRP box sections [5,13,14], I sections [6,15,16,17], wide-flange sections [4,8,14] and channel sections [14,18,19,20]. Furthermore, the end bearing with ground support (EG) and interior bearing with ground support (IG) were proposed by Wu et al. [5] to investigate the web-crippling behavior of GFRP box sections. It was found that the specimens under the IG loading configuration exhibited a larger load-bearing capacity than the ITF.

Apart from the different loading configurations, the mechanical properties, such as web-crippling and buckling of thin-walled structures, were significantly affected by the cracks [21,22,23,24,25] and openings [4,26,27,28]. Gand et al. [26] studied the web-crippling behavior of the perforated pultruded I sections under ITF, IG, ETF and EG loading configurations. It was found that the reduction of web-crippling strength can be up to 20% and 25% for specimens with circular and rectangular openings. The effect of the openings on the web-crippling behavior of pultruded wide-flange sections under ITF loading configuration was investigated by Haloi et al. [4]. The web-crippling strength of the specimens with a web perforation size of 0.8*h* (*h* was web depth) decreased by 41.1% compared to the intact specimens. Moreover, increasing the bearing length to web height ratio could significantly improve the web-crippling strength. Fernandes et al. [16,29] found that the web-crippling strength and stiffness could be improved by increasing the bearing length. Similar findings were reported by Wu et al. [20] for the pultruded GFRP channel sections under ITF and ETF loading configurations. Results showed that the bearing length exhibited a significant effect on the failure mode and load-displacement response, and the web-crippling capacity increased obviously with the increase in bearing length. Furthermore, Fernandes et al. [29] found that the pultruded GFRP I sections with different fiber layups exhibited similar distributions of transverse compressive strain under the same loading configuration, indicating that the fiber layup had a very limited effect on the effective bearing length.

Regarding the numerical studies on the web-crippling behavior, several finite element models were developed. A finite element model considering the shell element with the Tsai-Hill failure criterion was developed by Fernandes et al. [15] to investigate the pultruded GFRP I sections under ETF and ITF loading configurations. The Hashin failure criterion was frequently adopted to simulate the damage processes of the pultruded GFRP sections [6,30,31,32]. Based on the Hashin failure criterion and damage evolution law, Nunes et al. [6] developed a finite element model in Abaqus to simulate the progressive damage of the pultruded GFRP I sections under various loading conditions. It was found that the chamfer of the web-flange junction and the fracture energies had a significant influence on the web-crippling capacity. Gonilha et al. [33,34] developed an advanced progressive failure model comprising the damage progression and the constant stress beyond a limited strain for pultruded GFRP structures. The model was further validated through material level and web-crippling tests. More recently, the finite element models based on the fracture toughness were developed by Fernandes et al. [35] to investigate the effect of the instability and the material damage on the web-crippling behavior of the pultruded GFRP sections under ETF and ITF loading configurations.

The above studies were conducted on the GFRP sections under a normal service environment. Oskouei et al. [36] studied the web-crippling performance of the pultruded GFRP sections after subjecting them to wetting and drying cycles in seawater with temperatures of 40 °C and 60 °C. It was found that the seawater with high temperature and high chloride was the most aggressive environment for the sections. Although significant achievements have been made in understanding the web-crippling behavior of the pultruded GFRP and steel sections under a normal environment, few studies were reported on the web-rippling behavior under elevated temperatures [37]. It was found from the previous studies [38,39,40,41,42,43,44] the mechanical properties of the GFRP composites exhibited significant loss at elevated temperatures. To address this issue, the web-crippling experiments of the pultruded GFRP I sections at room temperature were conducted. Subsequently, the numerical model for simulating the web-crippling behavior of the pultruded GFRP I sections was developed and validated by the experiments. Finally, based on the developed model, the effects of elevated temperatures on the failure mode, damage profile, ultimate load and stress were presented and discussed.

## 2. Experimental Program at Room Temperature

### 2.1. Materials and Specimens

**Figure 1:**
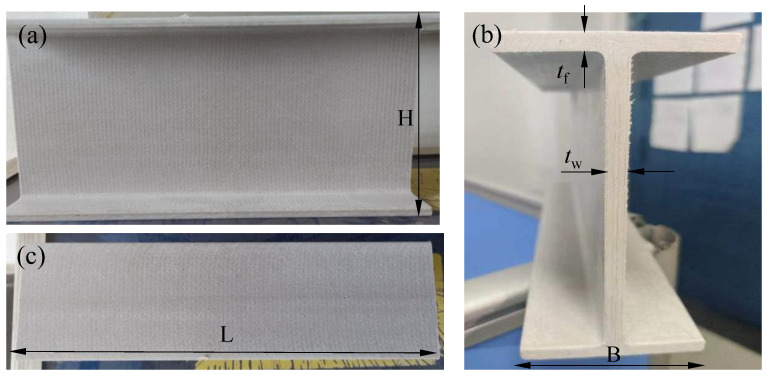
Appearance of the specimen: (**a**) front view; (**b**) side view and (**c**) top view.

As shown in Figure 1, the pultruded GFRP I section used in the experiment was supplied by the Nanjing Spare Composite Yizheng Company. The GFRP section consisted of the unidirectional glass rovings and glass mat layers embedded into the vinyl resin. The glass mat layer comprised a chopped strand mat (CSM) stitched with a 90° roving ply, which can be clearly observed in Figure 1a. Table 1 presents the geometry of the specimens. The width (B) and the height (H) of the section were 63.5 mm and 139.7 mm, respectively. The length (L) of the specimens was determined to be 300 mm, which was greater than twice the section height. The web and flange shared the same thickness of 6.35 mm. The mechanical properties of the GFRP section were obtained from the manufacturer. The longitudinal tensile strength and modulus were measured according to ASTM D638. The transverse compressive strength, modulus and the longitudinal compressive strength were measured according to ASTM D695. Moreover, according to ASTM D2344, the short-beam test was conducted to obtain the interlaminar shear strength. The in-plane shear modulus was adopted from reference [16], in which a similar GFRP I section (I120) was tested. Table 2 shows the elastic properties, while Table 3 shows the strength properties.

### 2.2. Experimental Instruments and Set-Up

**Figure 2:**
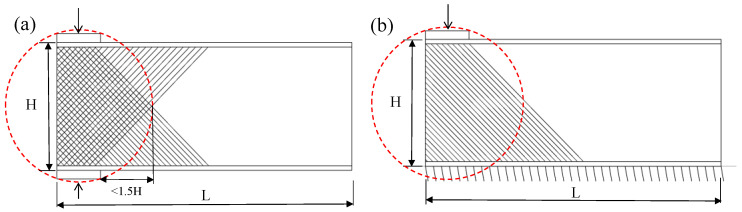
Loading configurations of (**a**) ETF and (**b**) EG.

As shown in Table 1 and Figure 2, a total of four specimens were designed and tested considering the ETF and the EG loading configurations. Two repeated specimens were tested for each loading configuration. As shown in Table 1, the label of the specimen consists of four parts. The first part, ETF or EG, denotes the loading configuration. The second part, 139.7, denotes the height of the cross-section. The third part, b50, denotes the bearing length of 50 mm. The fourth part, 1 or 2, denotes the index of the repeated specimens.

**Figure 3:**
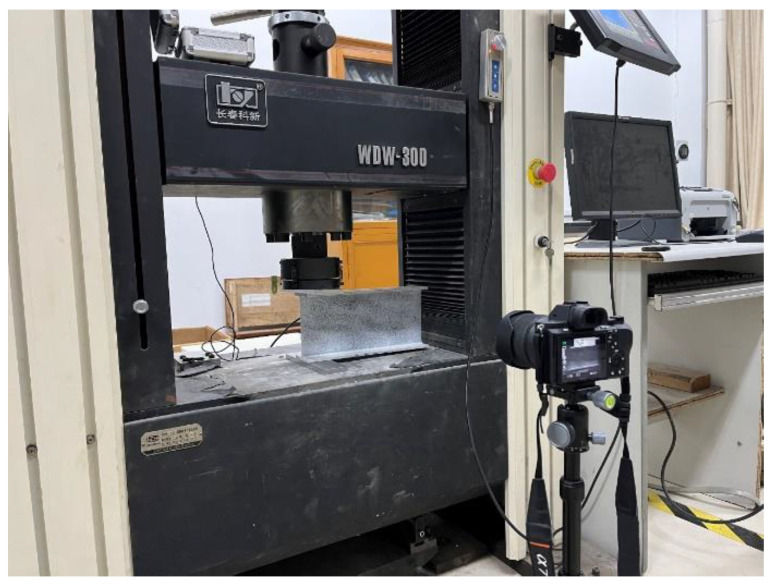
Test set-up of specimens under ETF loading configuration.

The test set-up for the web-crippling experiments is shown in Figure 3. In order to measure the displacement and the strain fields, the digital image correlation (DIC) technique was adopted in the experiments. The specimen was sprayed with speckles using white and black paints. The specimen was seated on the WDW-300 universal test machine. For ETF loading configuration, two steel bearing plates were respectively placed on the top and bottom flanges at the end. For the EG loading configuration, only one steel bearing plate was placed on the top flange at the end. The load was transmitted from the load cell of the test machine to the upper steel bearing plate. The specimen was loaded by the displacement control at a speed of 1 mm/min. A video camera was used to record the pictures of the specimen at a speed of 1 Hz. The pictures were then input to the Gom Correlate software for conducting the DIC post-analysis to obtain the displacement and strain fields.

## 3. Experimental Results and Discussion

### 3.1. Experimental Observation and Failure Modes

**Figure 4:**
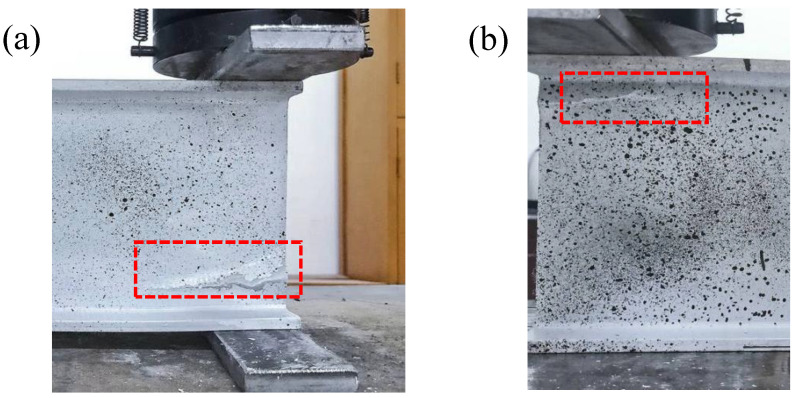
Failure modes of the specimens (**a**) ETF139.7-b50-2; (**b**) EG139.7-b50-2.

For specimens under ETF loading configuration, no obvious deformation was found within the specimen at the initial loading stage. The web-crippling deformation gradually increased with the increase of the load. A clear web-crippling deformation was found before the failure of the specimen ETF139.7-b50-1. Subsequently, the specimen failed due to the local web crushing, presenting a longitudinal crack near the lower web-flange junction. Similarly, the specimen ETF139.7-b50-2 failed due to the web crushing with a longitudinal crack near the lower web-flange junction, as shown in Figure 4a. The web crushing failure was consistent with the failure mode observed in the pultruded GFRP I sections with a height of 100 mm under ETF loading configuration [16]. For specimens under EG loading configuration, the web-crippling deformation is smaller than that of the specimens under ETF loading configuration before the failure. The specimens under EG loading configuration exhibited web crushing with a longitudinal crack found near the web-flange junction (Figure 4b).

### 3.2. Load-Displacement Response

**Figure 5:**
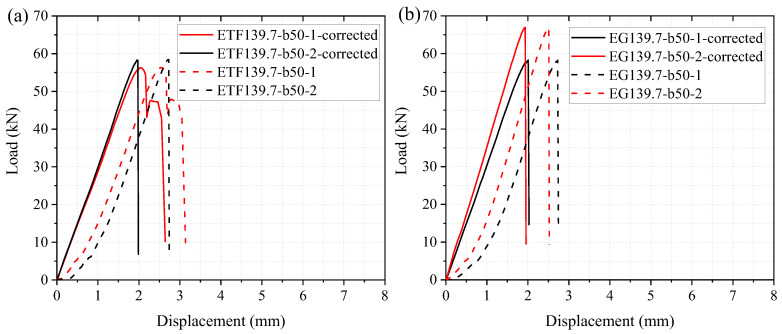
Load-displacement curves of specimens under (**a**) ETF and (**b**) EG loading conditions.

The load versus displacement relationships were presented as the dashed lines in Figure 5. At the initial stage, the load increased slowly with the increased displacement. This lower stiffness (slope of the load-displacement curve) was due to the adjustment of the test set-up. Subsequently, the load increased approximately linearly up to the ultimate load, followed by a sudden drop in the load. In order to obtain the true stiffnesses, the load-displacement curves were corrected by the ‘Toe compensation’ method according to ASTM D790 [45]. It can be seen from Figure 5 that the corrected curves of the repeated specimens exhibited similar ultimate load and stiffness. The average ultimate load of the specimens under the ETF loading condition is 57.4 kN, which was slightly lower than that under the EG loading condition (62.6 kN). Moreover, it can be seen from Figure 5 that the displacements at failure (ultimate load) for all the specimens are similar (2.6 mm and 2 mm for uncorrected and corrected curves, respectively). This is consistent with the experimental results found by Nunes et al. [6], in which the average displacement (uncorrected) at the failure of the specimens under ETF loading conditions was approximately 3 mm.

### 3.3. Strain Analysis

**Figure 6:**
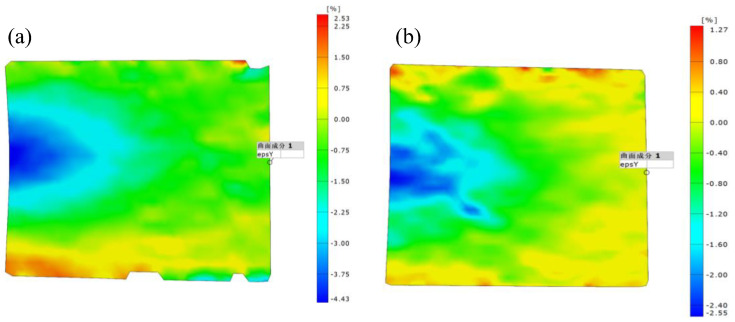
Transverse strains of the specimens (**a**) ETF139.7-b50-1 and (**b**) EG139.7-b50-2.

Based on the recorded pictures, the strain fields were obtained by the DIC analysis. Figure 6 presents the distributions of the transverse strain of the specimens at the ultimate load. It can be seen that most of the region below the steel bearing plate exhibited non-uniform distribution of the transverse compressive strain. The largest transverse strain was concentrated in the middle of the web due to the web-crippling deformation. The transverse compressive strain gradually decreased with the increase of the distance from the end. Moreover, the transverse tensile strain was observed at the bottom of the end. This resulted from the web crushing near the lower web-flange junction. As shown in Figure 6b, the distribution of the transverse strain of the specimen under the EG loading configuration is similar to the one under the ETF loading configuration. However, the area of the concentrated transverse compressive strain (blue) of specimen ETF139.7-b50-1 was much smaller than that of specimen EG139.7-b50-2. Furthermore, the transverse strain at the bottom of the end of specimen ETF139.7-b50-1 was significantly higher than that of specimen EG139.7-b50-2. This was attributed to the shorter bearing length (50 mm) of specimen ETF139.7-b50-1 than that (300 mm) of specimen EG139.7-b50-2. Furthermore, the transverse strain at the top of the end of specimen EG139.7-b50-2 was much smaller than that at the bottom of the end, where the web crushing was found (Figure 4b).

## 4. Numerical Modelling of Web-Crippling Behavior at Elevated Temperatures

### 4.1. Validation of the Numerical Model at Room Temperature

#### 4.1.1. Material Properties and Finite Element Mesh

**Figure 7:**
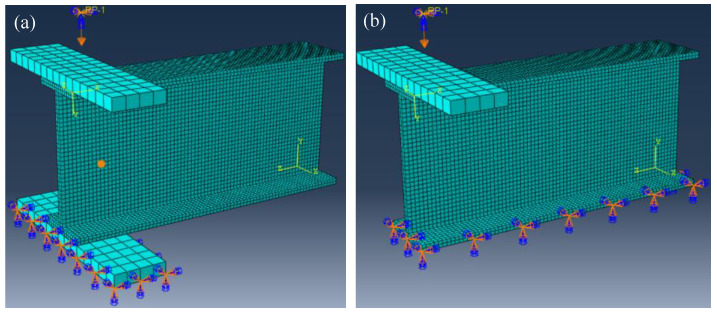
Geometry, mesh and boundary condition of the specimen under (**a**) ETF and (**b**) EG loading conditions.

In this study, the finite element software Abaqus was adopted to investigate the mechanical behavior of the pultruded GFRP I sections under the ETF or the EG loading configurations. The elastic and strength properties of the orthotropic GFRP material in Table 2 and Table 3 were adopted in the finite element modelling. The steel bearing plate, with an elastic modulus of 210 GPa and a Poisson’s ratio of 0.3, was simulated as the ideal elastomer. The continuum shell element (reduced integration SC8R) was adopted to mesh the GFRP I section with a length of 300 mm, while the 8-note C3D8R element with a size of 12.7 mm was used to mesh the steel bearing plate. Previous studies [46,47,48] showed that the mesh size might affect the accuracy of the finite element model. To evaluate the mesh size sensitivity of the finite element model, four mesh sizes, i.e., 2.5 mm, 4 mm, 6.5 mm and 10 mm, were adopted for the GFRP section. The mesh details of the finite element model with a mesh size of 4 mm are presented in Figure 7.

#### 4.1.2. Load and Boundary Conditions

As shown in Figure 7, the steel bearing plate was connected to the flange of the GFRP section using the “Tie” constraint. For specimens under ETF loading configuration, all the displacements and the rotations of the bottom face of the lower steel bearing plate were fixed (U1 = U2 = U3 = UR1 = UR2 = UR3 = 0). The transverse displacement was applied to the reference point, which was coupled with the upper steel bearing plate. For specimens under EG loading configuration, the bottom face of the lower GFRP flange was fixed while the displacement was applied on the reference point (the upper steel plate).

#### 4.1.3. Damage Initiation Criterion and Damage Evolution Law

In this study, the Hashin failure criterion [49] was adopted to simulate the damage initiation of web-crippling of the pultruded GFRP I sections. The Hashin failure criterion consists of four failure indexes, including the fiber tension index *F*_ft_, fiber compression index *F*_fc_, matrix tension index *F*_mt_ and matrix compression index *F*_mc_, as expressed as:

(1) $$F_{\mathrm{ft}}=\frac{\sigma_{1}^{2}}{S_{t,1}^{2}}+\alpha \frac{\sigma_{12}^{2}}{S_{12}^{2}}<1.0$$

(2) $$F_{\mathrm{fc}}=\frac{\sigma_{1}^{2}}{S_{c,1}^{2}}<1.0$$

(3) $$F_{\mathrm{mt}}=\frac{\sigma_{2}^{2}}{S_{t,2}^{2}}+\frac{\sigma_{12}^{2}}{S_{12}^{2}}<1.0$$

(4) $$F_{\mathrm{mc}}=\frac{\sigma_{2}^{2}}{4S_{23}^{2}}+\left(\frac{S_{c,2}^{2}}{4S_{23}^{2}}-1\right)\frac{\sigma_{2}}{S_{c,2}}+\frac{\sigma_{12}^{2}}{S_{12}^{2}}<1.0$$

where *σ_i_* and *S_i_* denote the stress and the strength in *i* direction (*i* = 1 denotes the longitudinal, *i* = 2 denotes the transverse, *i* = 12 denotes the longitudinal in-plane shear, and *i* = 23 denotes the transverse shear); the subscripts *t* and *c* denote the tensile and compressive strengths, respectively; *α* denotes the factor for considering the contribution of longitudinal shear stress to the fiber tension index.

**Figure 8:**
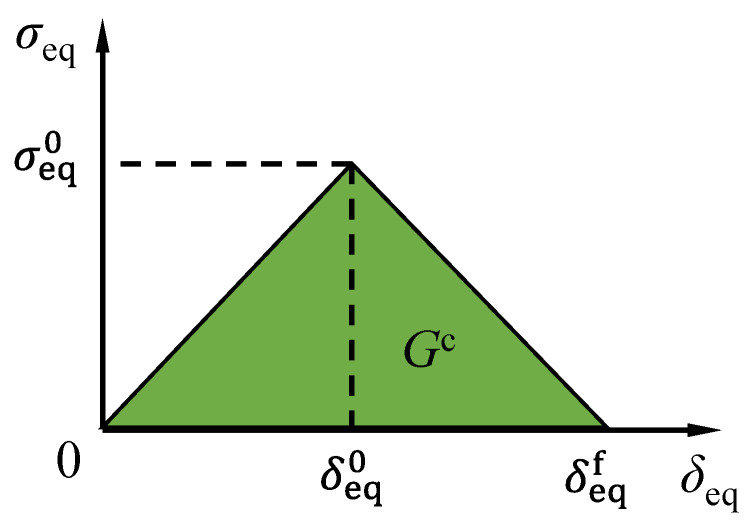
Equivalent stress-equivalent displacement relationship for damage evolution.

As shown in Figure 8, the equivalent stress-equivalent displacement was adopted in Abaqus to simulate the progressive failure process of the pultruded GFRP sections. The damage variable *d* was expressed as:

(5) $$d=\frac{\delta_{\mathrm{eq}}^{f}}{\delta_{\mathrm{eq}}}\cdot \frac{\left(\delta_{\mathrm{eq}}-\delta_{\mathrm{eq}}^{0}\right)}{\left(\delta_{\mathrm{eq}}^{f}-\delta_{\mathrm{eq}}^{0}\right)}$$

where *δ*_eq_ is the equivalent displacement; $\delta_{\mathrm{eq}}^{0}$ is the equivalent displacement when the damage initiates; $\delta_{\mathrm{eq}}^{f}$ is the equivalent displacement when the material is totally damaged. It should be noted that the damage was initiated when the Hashin failure index reached 1 while the equivalent displacement reached $\delta_{\mathrm{eq}}^{0}$. Regarding the fracture energies, it should be noted that significant underestimations (20.8~22.8%) of the ultimate load were found when using the recommended 10 times (9.48 N/mm) of the basic value of the transverse fracture energy [6]. Moreover, the fracture experiments of GFRP composites showed that the transverse compressive fracture energies were in the range of 40 to 67 N/mm, depending on the fiber layups. Therefore, in this study, an intermediate value of 28.44 N/mm (30 times the basic value of fracture energy from reference [6]) for the transverse fracture energy was adopted in this study, as shown in Table 4. The detailed calculation processes of the equivalent displacements and the equivalent stresses for fiber tension, fiber compression, matrix tension and matrix compression can be found in the Abaqus document [50].

Accordingly, the damage variables were calculated as follows:

(6) $$d_{f}=\{\begin{array}{c} d_{\mathrm{ft}}\;\;\mathrm{when}\;\sigma_{1}\geq 0\\ d_{\mathrm{fc}}\;\;\mathrm{when}\;\sigma_{1}<0 \end{array}$$

(7) $$d_{m}=\{\begin{array}{c} d_{\mathrm{mt}}\;\;\mathrm{when}\;\sigma_{2}\geq 0\\ d_{\mathrm{mc}}\;\;\mathrm{when}\;\sigma_{2}<0 \end{array}$$

(8) $$d_{\mathrm{ps}}=1-\left(1-d_{\mathrm{ft}}\right)\left(1-d_{\mathrm{fc}}\;\right)\left(1-d_{\mathrm{mt}}\right)\left(1-d_{\mathrm{mc}}\right)$$

where *d*_f_ is the damage variable of fiber; *d*_ft_ and *d*_fc_ are the damage variables of fiber tension and fiber compression, respectively. *d*_m_ is the damage variable of matrix; *d*_mt_ and *d*_mc_ are the damage variables of matrix tension and matrix compression, respectively. *d*_ps_ is the damage variable of in-plane shear, depending on the individual damage variables of fiber (matrix) in tension (compression). According to Equations (6)–(8), the stress-strain relationship considering the material damage can be obtained:

(9) σ=C(d)ε

where ***σ*** is the stress tensor, ***ε*** is the strain tensor; ***C***(*d*) is the stiffness matrix considering material damage:

(10) $$C\left(d\right)=\frac{1}{D}\left[\begin{array}{ccc} \left(1-d_{f}\right)E_{1} & \left(1-d_{f}\right)\left(1-d_{m}\right)\upsilon_{21}E_{1} & 0\\ \left(1-d_{f}\right)\left(1-d_{m}\right)\upsilon_{12}E_{2} & \left(1-d_{m}\right)E_{2} & 0\\ 0 & 0 & D\left(1-d_{\mathrm{ps}}\right)G \end{array}\right]$$

where *E* is the elastic modulus; *G* is the shear modulus; *ν* is the Poisson’s ratio; *D* = 1 − *ν*_12_*ν*_21_(1 − *d*_f_)(1 − *d*_m_).

#### 4.1.4. Mesh Sensitivity and Validation of the Finite Element Model

**Figure 9:**
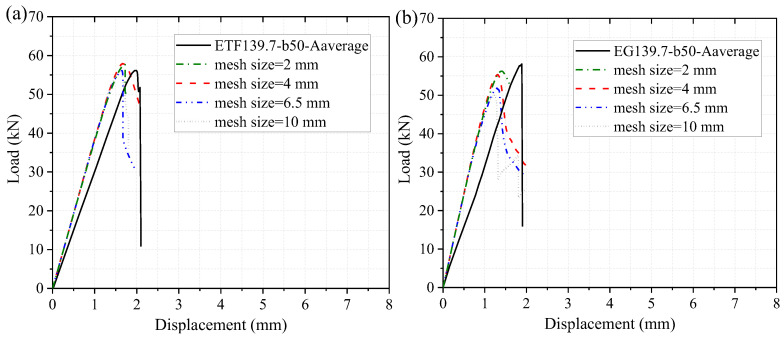
The experimental and numerical load-displacement curves of the specimens under (**a**) ETF and (**b**) EG loading conditions at room temperature.

Figure 9 shows the numerical load-displacement curves of the specimens at room temperature (20 °C) considering different mesh sizes. With the decrease in the mesh size, the ultimate load slightly increased while the stiffness remained unchanged. Considering the computational efficiency and the accuracy, the mesh size of 4 mm was adopted in the following investigations of the web-crippling behavior at room and elevated temperatures. Figure 9 presents the comparison of experimental and numerical load-displacement curves. It should be noted that the presented experimental curve under each loading configuration was obtained based on the average data of the two repeated specimens. For the specimen under ETF loading configuration, the simulated load increased linearly up to the initial damage at the web-flange junction near the steel bearing plate. Subsequently, the simulated load increased slightly up to the failure of the specimen, followed by a sudden drop in the load. Good agreements were found between the experimental and numerical ultimate loads. The simulated ultimate load of the specimen (mesh size of 4 mm) under ETF loading configuration was very close to the experimental value, while the simulated ultimate load of the specimen under EG loading configuration was 4.6% lower than the experimental value. Moreover, it was found that the experimental stiffness (slope of the load-displacement curve) was slightly lower than the numerical value. This can be understood since the experimental displacement was measured from the cross-beam of the universal machine rather than the steel bearing plate.

**Figure 10:**
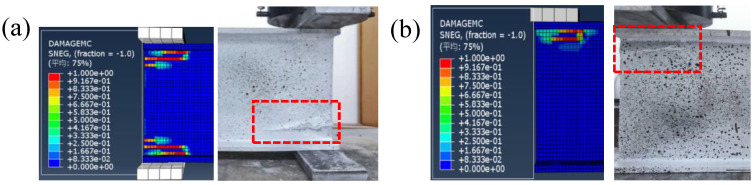
Comparison between the experimental failure mode and numerical damage pattern at room temperature.

Figure 10 shows the comparisons between the experimental failure modes and numerical damage distributions of matrix compression (mesh size = 4 mm) under ETF and EG loading configurations at room temperature. Regarding the ETF loading configuration, the predicted damage of matrix compression was concentrated at the web-flange junction zone below or above the bearing plate. Moreover, the damage area at the edge of the section was significantly larger than that at the interior. This is consistent with the cracking pattern observed on the specimen ETF139.7-b50-2 in Figure 10a. As for the specimen under EG loading configuration, the predicted damage of matrix compression was only observed at the web-flange junction below the bearing plate rather than above the ground. It can be seen that good agreements were found between the experimental cracking patterns and the predicted damage zones for all loading configurations, indicating the numerical model can accurately predict the web-crippling failure modes.

### 4.2. Numerical Model at Elevated Temperatures

#### 4.2.1. Temperature-Dependent Material Properties

**Figure 11:**
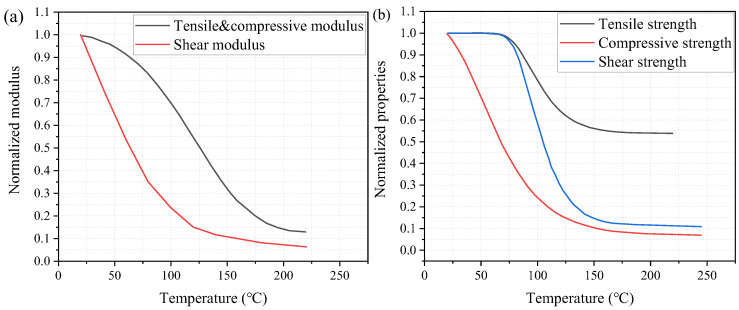
Temperature-dependent material properties: (**a**) modulus; (**b**) strength.

To consider the effect of elevated temperatures on the web-crippling behavior of the pultruded GFRP I sections, the temperature-dependent material properties were adopted in the numerical modelling, as shown in Figure 11. The elastic modulus was calculated by using the rule of mixture [40] while the shear modulus was obtained by using the inverse rule of mixture [51]. Figure 11b presents the temperature-dependent normalized tensile, compressive and shear strengths adopted from reference [38], where similar GFRP sections were used in the material test under elevated temperatures.

#### 4.2.2. Validation of the Developed Numerical Model Considering Temperature-Dependent Material Properties

To verify the feasibility of the extension of the developed finite element model from room temperature to elevated temperatures, the 10° off-axis tension (shear) and uniaxial tension tests of the pultruded GFRP laminates at temperatures 20, 100, 140 and 220 °C were adopted [40]. The pultruded GFRP laminates with the dimensions of 350 mm × 30 mm × 10 mm and 400 mm × 20 mm × 10 mm were used for the 10° off-axis tension test and the uniaxial tension test, respectively. All the specimens were loaded in tension at a displacement rate of 2 mm/min. In the numerical model, the mechanical properties at room temperature were adopted from references, while those at high temperatures were calculated based on the normalized modulus and strengths in Figure 11. It should be noted that the experimental tensile strength at 220 °C was adopted in the numerical model since it cannot be well predicted by the normalized curve of tensile strength in Figure 11b. Steel tabs were placed on the ends of the specimen to avoid failure in the grips. The same element type (continuum shell element SC8R), damage initiation criterion and damage evolution used in the model at room temperature were adopted for the model at elevated temperature.

**Figure 12:**
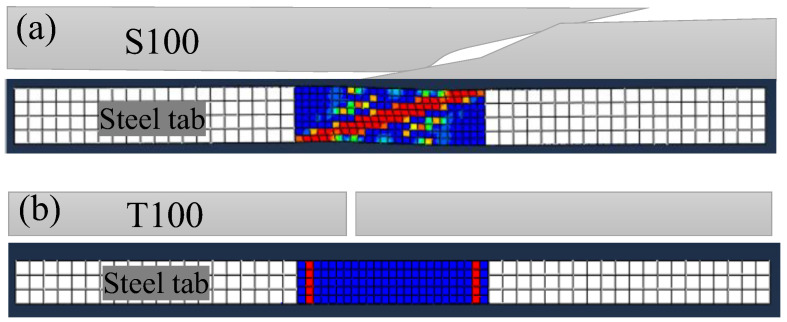
Validation of the developed numerical model by the experimental failure modes at 100 °C: (**a**) 10° off-axis tension (shear); (**b**) uniaxial tension [40].

Table 5 presents the comparison between the experimental ultimate loads *P*_exp_ and the numerical predictions *P*_num_. It can be seen that good agreements were found between both results for all the tested specimens. The average value of the *P*_num_/*P*_exp_ is 0.97, with a coefficient of variation of 0.07. The comparison of the failure modes at 100 °C obtained from the experiments with those obtained from the numerical model is depicted in Figure 12. Good agreement was achieved between the experimental and numerical failure modes for the specimen subjected to the 10° off-axis tension (shear) and uniaxial tension.

#### 4.2.3. Temperature-Dependent Load-Displacement Responses

**Figure 13:**
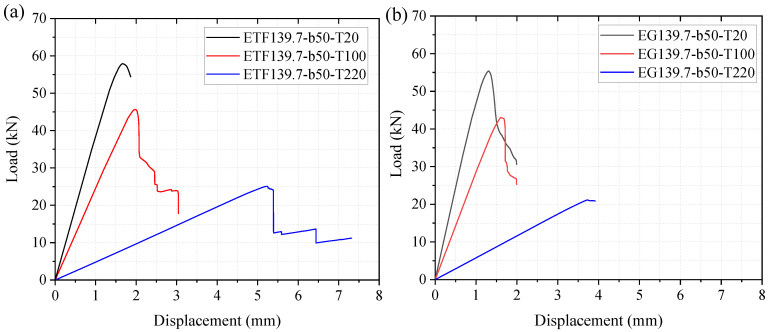
The influence of elevated temperatures on the load-displacement curve under (**a**) ETF and (**b**) EG loading configurations.

The numerical load-displacement curves of the pultruded GFRP specimens at 20, 100 and 220 °C are presented in Figure 13. The load-displacement curves of the specimens at 100 and 220 °C exhibited more progressive damage than the specimens at room temperature, except for the specimen under EG loading conditions at 220 °C. The ultimate load (web-crippling strength) reduced obviously with the increase in temperature. For specimens under ETF loading configuration, the ultimate loads at 100 and 220 °C were reduced by 21% and 57%, respectively, compared to the ultimate load at room temperature, whereas the ultimate loads of specimens under EG loading configuration at 100 and 220 °C reduced by 22% and 62%, respectively. Moreover, the stiffness of the specimens significantly reduced with the increasing temperature. For the specimens under ETF loading configuration, the elastic stiffness decreased by 37% and 87% at 100 and 220 °C, respectively, compared to that at room temperature. For the specimens under EG loading configuration, a larger reduction in elastic stiffness (41% at 100 °C and 88% at 220 °C) was found. Hence, the low web crippling strength and stiffness at elevated temperatures should be addressed in the design of the pultruded GFRP sections when subjected to concentrated mechanical and thermal loadings.

#### 4.2.4. Progressive Web-Crippling Failure Process at Elevated Temperatures

**Figure 14:**
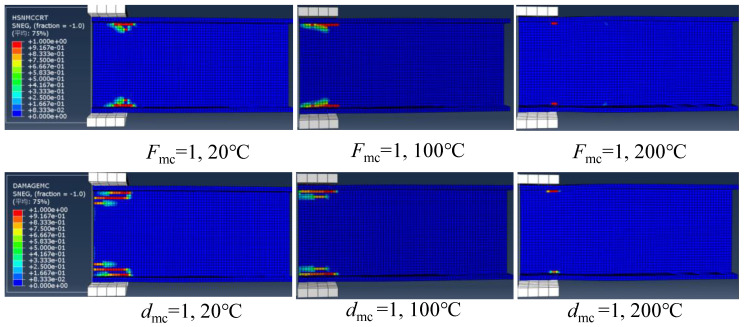
The matrix compressive damage distributions in case of ETF at different temperatures under the initial damage state and ultimate load state.

Based on the numerical modelling with Hashin criterion, it was found that the damage initiated by the matrix compression. Figure 14 presents the matrix compressive damage distributions of specimens under ETF loading configuration at 20, 100 and 220 °C. It can be seen that the matrix compressive damage initiated (i.e., Hashin failure index *F*_mc_ = 1) at the web-flange junction near the corner of the steel bearing plate. As the temperature increased, the damage area gradually reduced. At 220 °C, the Hashin failure index of only one element near the steel bearing plate reached 1. Figure 14 also presents the matrix compressive damage distributions at the ultimate load (i.e., damage index *d*_mc_ = 1). Under the same temperature, the damage distribution at the ultimate load significantly extended compared to the initial damage state due to the progressive damage evolution. At the ultimate load state, a similar reduction in damage area compared to the initial damage state was found with the increase of temperature.

**Figure 15:**
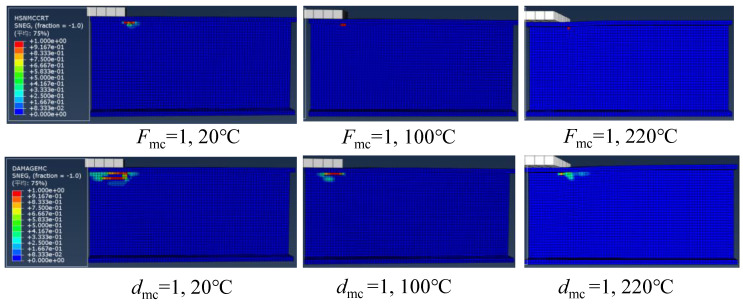
The matrix compressive damage distributions in the case of EG at different temperatures under the initial damage state and ultimate load state.

Regarding the specimens under EG loading configuration, Figure 15 depicts the matrix compressive damage distributions under different temperatures at the initial damage state and ultimate load state. The damage initiated at the web-flange junction near the corner of the upper flange and significantly propagated toward the end. As the temperature increased, the damage area gradually reduced. Moreover, unlike the symmetrical damage distributions under the ETF loading configuration, no damage was observed in the web-flange junction above the ground (base) under the EG loading configuration.

#### 4.2.5. Temperature-Dependent Stress Responses

**Figure 16:**
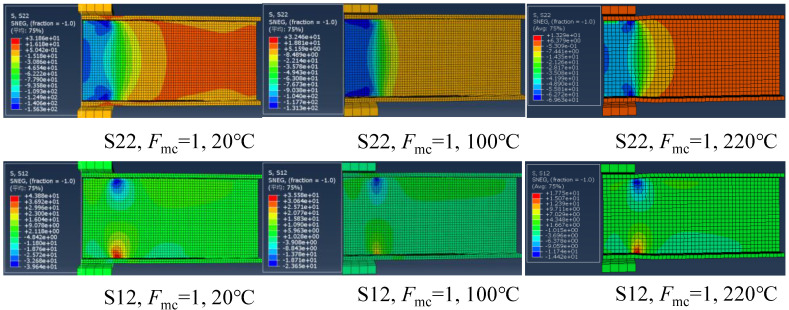
The transverse compressive stress and in-plane shear stress distributions in the case of ETF under different temperatures under initial damage state.

**Figure 17:**
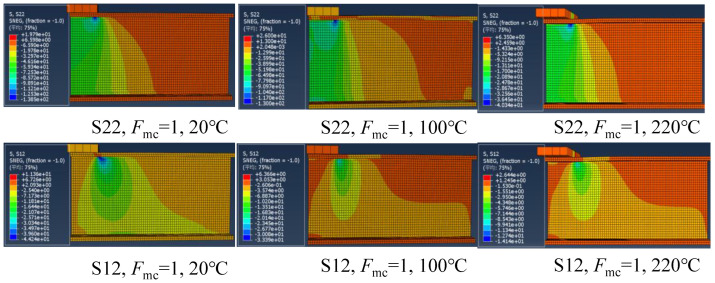
The transverse compressive stress (S22) and in-plane shear stress (S12) distributions in the case of EG at different temperatures under initial damage state.

Figure 16 presents the transverse compressive stress (S22) and in-plane shear stress (S12) distributions of the specimens at the initial damage state under the ETF loading configuration and different temperatures. The stress distribution is generally symmetrical due to the symmetry of the loading configuration. At room temperature, the maximum transverse compressive stress (156 MPa) at the web-flange junction near the steel bearing plate is slightly lower than its strength (158.5 MPa), whereas the in-plane shear stress (39.6 MPa) exceeded the in-plane shear strength (31.9 MPa). A similar trend was found for the specimens at 100 and 220 °C. This indicated that the initial damage was triggered by exceeding the in-plane shear strength and independent of the temperature. Moreover, as the temperature increased, the transverse compressive stress and in-plane shear stress reduced significantly reduced. Figure 17 shows the transverse compressive stress (S22) and in-plane shear stress (S12) distributions of the specimens at the initial damage state under the EG loading configuration and different temperatures. Unlike the symmetrical stress distribution of the specimens under ETF loading configuration, the stress distribution is generally unsymmetrical. The stress was concentrated at the web-flange junction near the corner of the upper steel bearing plate and gradually decreased along the transverse (downward) and longitudinal (rightward) directions. Moreover, the specimens under EG loading configuration were also triggered by exceeding the in-plane shear strength at different temperatures.

## 5. Conclusions

In this study, web-crippling experiments were conducted on the pultruded GFRP I sections under the end-two-flange (ETF) and the end bearing with ground support (EG). The finite element model was developed and verified by the experiments at room temperature and elevated temperatures. Finally, the developed mode was used to investigate the influences of elevated temperatures on web-crippling behavior. Based on the experimental and numerical results, the following conclusions can be drawn:

(1) The initial damage of the pultruded GFRP I sections was triggered by exceeding the shear strength at the web-flange junction near the corner of the steel bearing plate and independent of the elevated temperatures and loading configurations. The pultruded GFRP I sections failed by the web crushing with a longitudinal crack propagated from the web-flange junction near the corner of the steel bearing plate.
(2) At room temperature, no significant difference in the ultimate load (web-crippling strength) of the pultruded GFRP I sections was found between the end-two-flange and end-bearing-with-ground-support loading configurations. The stiffness and displacement at the failure of the specimen under the end-two-flange loading configuration were close to those under the end-bearing-with-ground-support loading configuration.
(3) A finite element model based on the Hashin failure criterion, damage evolution law and the temperature-dependent material properties was developed to simulate the web-crippling behavior of the pultruded GFRP I sections under elevated temperatures. The model was verified with web-crippling experiments at room temperature as well as the 10° off-axis tension and the uniaxial tension experiments at elevated temperatures. Good agreements were found between the experimental and numerical ultimate loads and failure modes.
(4) The ultimate load decreased obviously with the increasing temperature. For specimens under the end-two-flange loading configuration, the ultimate loads at 100 and 220 °C were reduced by 21% and 57%, respectively, whereas the ultimate loads of the specimens under the end-bearing-with-ground-support loading configuration at 100 and 220 °C were reduced by 22% and 62%, respectively. Moreover, the stiffness reduced faster than the ultimate load with the increase in temperature. As an example, For the specimens under the end-two-flange loading configuration, the elastic stiffness decreased by 37% and 87% at 100 and 220 °C, respectively, compared to that at room temperature.

## Figures and Tables

**Table 1:** Specimens’ geometry details (mm).

Specimen	Length (*L*)	Height (*H*)	Width (*B*)	Flange Thickness (*t*_f_)	Web Thickness (*t*_w_)	H/B (-)
ETF139.7-b50-1	300	139.7	63.5	6.35	6.35	2.2
ETF139.7-b50-2	300	139.7	63.5	6.35	6.35	2.2
EG139.7-b50-1	300	139.7	63.5	6.35	6.35	2.2
EG139.7-b50-2	300	139.7	63.5	6.35	6.35	2.2

**Table 2:** Elastic properties of the GFRP material.

*E*_1_ (GPa)	*E*_2_ (GPa)	*G*_12_ (GPa)	*G*_13_ (GPa)	*G*_23_ (GPa)	*v* (-)
25.5	12.1	3.9	3.9	1.6	0.266

*E* is the elastic modulus; *G* is the shear modulus; *ν* is the Poisson’s ratio. The subscripts 1 denotes the longitudinal direction while 2 and 3 denote the transverse direction.

**Table 3:** Strength properties of the GFRP material (MPa).

*S*_1,t_	*S*_1,c_	*S*_2,t_	*S*_2,c_	*S*_12_	*S*_23_
336.7	319.6	158.5	158.5	31.9	31.9

*S* denotes the strength. The subscripts *t* and c represent the tensile and compressive strengths, respectively. The value of *S*_2,c_ was adopted for *S*_2,t_**_._** The value of *S*_23_ was adopted for *S*_12_**_._**

**Table 4:** Fracture energies used in the modelling (N/mm).

Fiber Tension *G*_ft_	Fiber Compression *G*_fc_	Matrix Tension *G*_mt_	Matrix Compression *G*_mc_
71.4	158.4	12.72	28.44

**Table 5:** Comparison of numerical and experimental temperature-dependent ultimate loads of the pultruded GFRP laminates subjected to 10° off-axis tension (shear) and uniaxial tension.

Specimens	*P*_num_ (kN)	*P*_exp_ (kN)	*P*_num_/*P*_exp_
S20	52.2	51.1	1.02
S100	32.1	30.4	1.06
S140	19.4	19.7	0.98
S220	6.7	6.5	1.03
T20	63.7	68.6	0.93
T100	46.2	49.8	0.93
T140	37.4	41.3	0.91
T220	15.5	18.1	0.86
Average			0.97
COV			0.07

S and T denote the 10° off-axis tensile and the uniaxial tensile tests, respectively; the number denotes the temperature in °C.

## Data Availability

The data presented in this study are available on reasonable request from the corresponding author.

## References

[1] Ferdous W., Bai Y., Ngo T.D., Manalo A., Mendis P. (2019). New advancements, challenges and opportunities of multi-storey modular buildings–A state-of-the-art review. Eng. Struct..

[2] Zhang L., Liu W., Wang L., Ling Z. (2019). Mechanical behavior and damage monitoring of pultruded wood-cored GFRP sandwich components. Compos. Struct..

[3] Hollaway L.C. (2010). A review of the present and future utilisation of FRP composites in the civil infrastructure with reference to their important in-service properties. Constr. Build. Mater..

[4] Haloi J., Borsaikia A.C., Singh K.D. (2021). Web crippling behaviour of web perforated GFRP wide-flange sections subjected to interior-two-flange loading condition. Thin-Walled Struct..

[5] Wu C., Bai Y. (2014). Web crippling behaviour of pultruded glass fibre reinforced polymer sections. Compos. Struct..

[6] Nunes F., Silvestre N., Correia J.R. (2017). Progressive Damage Analysis of Web Crippling of GFRP Pultruded I-Sections. J. Compos. Constr..

[7] Young B., Hancock G.J. (2001). Design of Cold-Formed Channels Subjected to Web Crippling. J. Struct. Eng..

[8] Haloi J., Mushahary S.K., Borsaikia A.C., Singh K.D. (2021). Experimental investigation on the web crippling behaviour of pultruded GFRP wide-flange sections subjected to two-flange loading conditions. Compos. Struct..

[9] Borowicz D.T., Bank L.C. (2011). Behavior of Pultruded Fiber-Reinforced Polymer Beams Subjected to Concentrated Loads in the Plane of the Web. J. Compos. Constr..

[10] American Society of Civil Engineers (2002). Specification for the Design of Cold-Formed Stainless Steel Structural Members.

[11] Standards Australia International Limited (2001). Cold-Formed Stainless Steel Structures.

[12] EC3 (2006). Design of Steel Structures–Part 1.4: General Rules–Supplementary Rules for Stainless Steels.

[13] Chen Y., Wang C. (2015). Web crippling behavior of pultruded GFRP rectangular hollow sections. Compos. Part B Eng..

[14] Haloi J., Borsaikia A.C., Singh K.D. (2022). Mechanical characterization of pultruded GFRP channel, wide flange and rectangular hollow profiles. Novative Infrastruct. Solut..

[15] Fernandes L.A., Nunes F., Silvestre N., Correia J.R., Gonilha J. (2015). Web-crippling of GFRP pultruded profiles. Part 2: Numerical analysis and design. Compos. Struct..

[16] Fernandes L.A., Gonilha J., Correia J.R., Silvestre N., Nunes F. (2015). Web-crippling of GFRP pultruded profiles. Part 1: Experimental study. Compos. Struct..

[17] Chen Y., Wang C. (2015). Test on pultruded GFRP I-section under web crippling. Compos. Part B Eng..

[18] Zhang W., Chen Y. (2017). Tests on GFRP Pultruded Profiles with Channel Section Subjected to Web Crippling. Appl. Compos. Mater..

[19] Wu C., Zhang L.-T., Bai Y., Zhao X.-L. (2019). Web crippling behavior of pultruded GFRP channel sections under transverse bearing load. Compos. Struct..

[20] Wu C., Zhang L.-T., Tam L., Yan L., He L. (2020). Effect of bearing length on web crippling behavior of pultruded GFRP channel section. Compos. Struct..

[21] Duc N.D., Trinh T.D., van Do T., Doan D.H., Nguyen-Xuan H., Phung-Van P., Rabczuk T. (2018). On the Buckling Behavior of Multi-cracked FGM Plates. Proceedings of the International Conference on Advances in Computational Mechanics 2017.

[22] Doan D.H., Zenkour A.M., van Thom D. (2022). Finite element modeling of free vibration of cracked nanoplates with flexoelectric effects. Eur. Phys. J. Plus.

[23] Minh P.P., van Do T., Duc D.H., Duc N.D. (2018). The stability of cracked rectangular plate with variable thickness using phase field method. Thin-Walled Struct..

[24] Vedernikov A., Gemi L., Madenci E., Onuralp Özkılıç Y., Yazman Ş., Gusev S., Sulimov A., Bondareva J., Evlashin S., Konev S. (2022). Effects of high pulling speeds on mechanical properties and morphology of pultruded GFRP composite flat laminates. Compos. Struct..

[25] Gemi L., Madenci E., Özkılıç Y.O., Yazman Ş., Safonov A. (2022). Effect of Fiber Wrapping on Bending Behavior of Reinforced Concrete Filled Pultruded GFRP Composite Hybrid Beams. Polymers.

[26] Gand A.K., Mohammed M.H.M., Jarrouj S. (2020). Performance of perforated FRP stub beams subject to static transverse actions. Eng. Solid Mech..

[27] Madenci E., Özkılıç Y.O., Aksoylu C., Safonov A. (2022). The Effects of Eccentric Web Openings on the Compressive Performance of Pultruded GFRP Boxes Wrapped with GFRP and CFRP Sheets. Polymers.

[28] Aksoylu C., Özkılıç Y.O., Madenci E., Safonov A. (2022). Compressive Behavior of Pultruded GFRP Boxes with Concentric Openings Strengthened by Different Composite Wrappings. Polymers.

[29] Almeida-Fernandes L., Correia J.R., Silvestre N. (2021). Effect of fibre layup and bearing length on the web-crippling behaviour of pultruded GFRP profiles. Compos. Struct..

[30] Madenci E., Özkılıç Y.O., Gemi L. (2020). Experimental and theoretical investigation on flexure performance of pultruded GFRP composite beams with damage analyses. Compos. Struct..

[31] Madenci E., Onuralp Özkılıç Y., Gemi L. (2020). Buckling and free vibration analyses of pultruded GFRP laminated composites: Experimental, numerical and analytical investigations. Compos. Struct..

[32] Gemi L., Madenci E., Özkılıç Y.O. (2021). Experimental, analytical and numerical investigation of pultruded GFRP composite beams infilled with hybrid FRP reinforced concrete. Eng. Struct..

[33] Gonilha J.A., Silvestre N., Correia J.R., Tita V. (2021). and Almeida-Fernandes, L. Novel progressive failure model for quasi-orthotropic pultruded FRP structures: Application to compact tension and web-crippling case studies (Part II). Compos. Struct..

[34] Gonilha J.A., Silvestre N., Correia J.R., Tita V., Martins D. (2021). Novel progressive failure model for quasi-orthotropic pultruded FRP structures: Formulation and calibration of parameters (Part I). Compos. Struct..

[35] Almeida-Fernandes L., Silvestre N., Correia J.R. (2022). Fracture toughness-based models for web-crippling of pultruded GFRP profiles. Compos. Part B Eng..

[36] Oskouei A.V., Bazli M., Ashrafi H., Imani M. (2018). Flexural and web crippling properties of GFRP pultruded profiles subjected to wetting and drying cycles in different sea water conditions. Polym. Test..

[37] Zhou F., Young B. (2013). Web crippling behaviour of cold-formed duplex stainless steel tubular sections at elevated temperatures. Eng. Struct..

[38] Correia J.R., Gomes M.M., Pires J.M., Branco F.A. (2013). Mechanical behaviour of pultruded glass fibre reinforced polymer composites at elevated temperature: Experiments and model assessment. Compos. Struct..

[39] Correia J.R., Bai Y., Keller T. (2015). A review of the fire behaviour of pultruded GFRP structural profiles for civil engineering applications. Compos. Struct..

[40] Bai Y., Keller T. (2009). Modeling of strength degradation for fiber-reinforced polymer composites in fire. J. Compos. Mater..

[41] Mouritz A.P., Feih S., Kandare E., Mathys Z., Gibson A.G., Des Jardin P.E., Case S.W., Lattimer B.Y. (2009). Review of fire structural modelling of polymer composites. Compos. Part A Appl. Sci. Manuf..

[42] Zhang L., Liu W., Sun G., Wang L., Li L. (2017). Two-dimensional modeling of thermomechanical responses of rectangular GFRP profiles exposed to fire. Adv. Mater. Sci. Eng..

[43] Zhang L., Chen K., Liu W., Liu Y., Wang K., Ge W., Guo K. (2022). Fire performance of pultruded wood-cored GFRP sandwich components for building construction. Case Stud. Constr. Mater..

[44] Zhang L., Liu W., Wang L., Ling Z. (2020). On-axis and off-axis compressive behavior of pultruded GFRP composites at elevated temperatures. Compos. Struct..

[45] D20 Committee (2017). ASTM D790-17. Test Methods for Flexural Properties of Unreinforced and Reinforced Plastics and Electrical Insulating Materials.

[46] Atta M., Abu-Sinna A., Mousa S., Sallam H.E., Abd-Elhady A.A. (2022). Flexural behavior of functionally graded polymeric composite beams. J. Ind. Text..

[47] El-Sagheer I., Abd-Elhady A.A., Sallam H.E.D.M., Naga S.A., Sayed S. (2022). Flexural and fracture behaviors of functionally graded long fibrous polymeric composite beam-like specimens. Compos. Struct..

[48] El-Sagheer I., Abd-Elhady A.A., Sallam H.E.D.M., Naga S.A.R. (2021). An Assessment of ASTM E1922 for Measuring the Translaminar Fracture Toughness of Laminated Polymer Matrix Composite Materials. Polymers.

[49] Hashin Z. (1980). Failure Criteria for Unidirectional Fiber Composites. J. Appl. Mech..

[50] Abaqus 6.14 Documentation. http://130.149.89.49:2080/v6.14/index.html.

[51] Zhang L., Li Q., Cao D., Liu W., Wang K. (2022). In-plane shear properties of multi-axial pultruded composites at elevated temperatures. Acta Mater. Compos. Sin..

